# Environmental‐related variation of stoichiometric traits in body and organs of non‐native sailfin catfishes *Pterygoplichthys* spp

**DOI:** 10.1002/ece3.9483

**Published:** 2022-11-05

**Authors:** Hui Wei, Yanting Liang, Qiang Luo, Dangen Gu, Xidong Mu, Yinchang Hu

**Affiliations:** ^1^ Key Laboratory of Prevention and Control for Aquatic Invasive Alien Species (Ministry of Agriculture and Rural Affairs), Key Laboratory of Alien Species and Ecological Security (CAFS) Pearl River Fisheries Research Institute, Chinese Academy of Fisheries Science Guangzhou Guangdong China; ^2^ School of Marine Sciences Guangxi University Nanning Guangxi China; ^3^ College of Life Sciences and Food Engineering Yibin University Yibin Sichuan China

**Keywords:** allocation, calcium (Ca), Invasive fish, Loricariidae, nutrient limitation

## Abstract

Intraspecific variation in stoichiometric traits was thought to be an adaptive response to reduce the elemental imbalance between organism and diet in the habitat. Studying the spatial variation of stoichiometric traits of non‐native species and the factors contributing to the variation could help to better understand the invasion mechanism of non‐native fish. In this study, stoichiometric traits (i.e. carbon [C], phosphorus [P], calcium [Ca] and their ratios) variation in the body and organs of non‐native sailfin catfishes *Pterygoplichthys* spp. were investigated across 13 river sections in the main river basins of Guangdong province. The relationships between environmental factors and stoichiometric traits were analyzed using a general linear model and an information‐theoretic approach. A manipulated feeding experiment was conducted to investigate the impact of food quality on the stoichiometry of sailfin catfishes in a greenhouse. Sailfin catfishes exhibited considerable variability in body and organ elemental composition. Site identity was the main factor contributing to the variation, which could be explained by a combination of environmental factors including climate, diet quality, fish species richness and trophic status in the invaded rivers. Water chemistry (i.e. total nitrogen and phosphorus, ammonia nitrogen and soluble reactive phosphorus) contributed to the most variation of stoichiometric traits. Imbalances of P and Ca between sailfin catfishes and food resources varied among sampling sites, reflecting the spatial heterogeneity of nutrients limitation. Juvenile sailfin catfishes exhibited stoichiometric homeostasis (0 < 1/H < 0.25) for all elemental contents and ratios in the feeding experiment. These findings suggested variation in stoichiometric traits of sailfin catfishes might be attributed to the changes in elemental metabolism to cope with context‐specific environments. This study provided heuristic knowledge about environmental‐related variation in stoichiometric traits, which could enhance the understanding of the non‐native species' adaptation to resource fluctuation in the invaded ecosystems.

## Graphical abstract

We found sailfin catfishes can exhibit considerable variation in stoichiometric traits in response to the environmental dynamics of the invaded rivers. Water chemistry contributed to the most of variation of stoichiometric traits of sailfin catfishes. Imbalances of P and Ca between sailfin catfishes and food resources varied among sampling sites, reflecting the spatial heterogeneity of nutrient limitation. These findings suggested variation in stoichiometric traits might be attributed to changes in elemental metabolism to cope with context‐specific environments which directly or indirectly affected consumer performance, food resource quality and elemental imbalance.

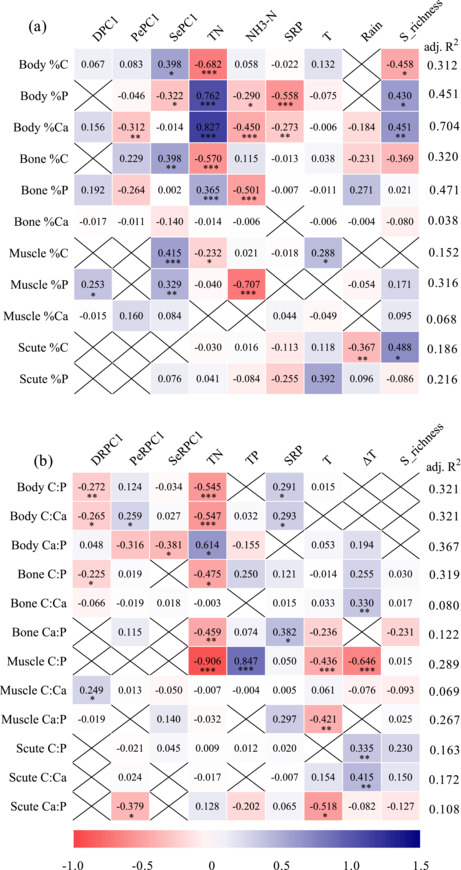

## 1 INTRODUCTION

Ecological stoichiometry provides a framework to explain the mismatch between the nutritional demand of organisms and the nutritional supply of environments in ecological interactions and ecosystem processes (Sterner & Elser, 2002). Elemental composition [e.g. carbon (C), nitrogen (N) and phosphorus (P)] can be reflected in the biomolecular composition of organisms (Sterner & Elser, 2002). For example, C is correlated with lipids or carbohydrates and N with protein (Sterner & Elser, 2002). P is related to nucleic acid (i.e. RNA and DNA) and is also correlated with apatite in bony structure and with phosphocreatine in the muscle of vertebrates (Breves & Schroder, 1991). In recent years, ecological stoichiometry has begun to move beyond the three important elements C, N and P(Hopper et al., 2021; Penuelas et al., 2019). For example, calcium (Ca) is also important for understanding ecological interactions between organisms and resource availability (Jones et al., 2020). In vertebrates, Ca mainly as tricalcium phosphate, is the key element of bony structures (e.g. bone and scute of fishes) and as calmodulin, also plays an important role in muscle contraction (Mahamid et al., 2010; Walsh, 1983). Stoichiometric traits, which are characterized by the biological process of the interactions between biologically relevant elements and environmental factors, were closely related to the growth, maturity and reproduction of the organisms (Sterner & Elser, 2002). Also, the variation in stoichiometric traits could affect population dynamics, nutrient cycling and species sorting (Capps & Flecker, 2013; Nakazawa, 2011; Teurlincx et al., 2017). Thus it is important to understand the factors contributing to the variation of stoichiometric traits, which can provide new insight into understanding the invasion mechanism of non‐native species (González et al., 2010).

Most animals can maintain fixed elemental ratios, by excreting excessive elements or concentrating limiting elements (Sterner & Elser, 2002). However, in recent years, intraspecific and interspecific variation of stoichiometric traits has been documented in various animal taxon including zooplankton (Declerck et al., 2015), invertebrate (Krist et al., 2016) and fish (El‐Sabaawi, Kohler, et al., 2012; El‐Sabaawi, Zandona, et al., 2012). Stoichiometric variability could be affected by intrinsic factors (i.e. organism traits) including body sizes, ontogenetic stages and sexes, which could be attributed to the balance between nutrient demands and availability throughout the life cycles (Boros et al., 2015). For example, juveniles and adults have different nutritional demands for rapid growth (i.e. P‐rich diets) at the early age and for reproduction (i.e. C‐rich diets) in the adult stage (Boros et al., 2015; Ebel et al., 2015). Also, differences in reproductive activity (i.e. nest guarding vs oviposition) and body size between females and males could drive sexual dimorphism in nutrient cycling and organismal stoichiometric traits (Ambus & Moody, 2019; Mozsar et al., 2019). Besides, biotic and abiotic environmental factors (i.e. external factors) could directly or indirectly influence the variation of stoichiometric traits including diet quality (Vrede et al., 2011), climate (Van Dievel et al., 2019), predators (Rinehart & Hawlena, 2020) and lake trophic state (Tuckett et al., 2016). Among them, food quality was the most studied factor, which directly affected the body elemental composition and excretion based on the nutrient demand of the organisms (e.g. Dalton et al., 2017;El‐Sabaawi, Kohler, et al., 2012; El‐Sabaawi, Zandona, et al., 2012). However, organisms might face complex environmental conditions in their habitats, in particular, under global change scenarios. Albeit the correlation between multiple environmental factors and stoichiometric traits has been well documented in plants, few studies have examined this for animals (El‐Sabaawi, Kohler, et al., 2012; El‐Sabaawi, Zandona, et al., 2012).

Nutrient allocation among growth‐ and reproduction‐related organs played a critical role for organisms to acclimate specific environments in their habitats (Van Noordwijk & De Jong, 1986; Wacker & Martin‐Creuzburg, 2007). To keep homeostasis, elements can transfer among organs when nutrients are limited (Breves & Schroder, 1991). For example, P and Ca contents of three spine stickleback (*Gasterosteus aculeatus*) could redistribute from lateral plates to other tissues of the unarmored form in freshwater habitats, while could reallocate from other tissues to lateral plates of armored form in marine habitats (Jeyasingh et al., 2014; Leal, Best, et al., 2017). Besides, nutrient allocation might be also affected by environmental factors due to the influence of environmental‐related trade‐offs among growth‐ and reproduction‐related organs (Van Noordwijk & De Jong, 1986). And also, C, N and P allocation between somatic and reproductive tissues could be restricted by food quality (Faerøvig & Hessen, 2003). For example, C‐related lipids (e.g. polyunsaturated fatty acids) were decreased in eggs and somatic tissues of *Daphnia magna* under poor food quality conditions (Wacker & Martin‐Creuzburg, 2007). However, there was a paucity of knowledge about the environmental‐related variation of elemental allocation among tissues, particularly among P and Ca‐rich morphological traits (e.g. bone and scute) (see Hopper et al., 2021).

Native to the Amazon river basin, sailfin catfishes *Pterygoplichthys* spp. in Family Loricariidae have invaded about 15 counties in tropic and subtropic regions (Orfinger & Douglas Goodding, 2018). These fishes, which are mainly *P. pardalis × P. disjunctivus* hybrids, were introduced into China since the 1990s and then established self‐sustaining populations in the main river basins of south China (Wei et al., 2017). Loricariid fishes were characterized by bony dermal plates which are modified elasmoid scales and originate from estrogen (Sire et al., 2009). For this reason, sailfin catfishes have higher P and Ca content relative to native fishes (Capps & Flecker, 2013). Hence their growth was likely to be limited by P (Hood et al., 2005). Moreover, environmental‐related variations of growth, maturity and reproduction of sailfin catfishes were also documented in the main river basins of south China, suggesting these fishes had different nutrient demands in response to the context‐specific environments in the invaded rivers (Wei et al., 2022). Since the intraspecific variation of stoichiometric traits could affect the fitness of organisms, the interactions between these traits and environmental factors could reflect how non‐native species acclimate to resource fluctuation in the invaded ecosystems (Snell‐Rood et al., 2015). In this respect, we hypothesized that stoichiometric traits of sailfin catfishes could vary across different populations and the variation might be attributed to the adaptive response to the context‐specific environments in the invaded rivers. To address this hypothesis, this study was designed to: (1) investigate spatial variation of stoichiometric traits of sailfin catfishes and test whether changes in environmental conditions were related to the variation; (2) examine spatial variation of elemental allocation in different body parts to examine whether elements could be reallocated among organs in changing environments; and (3) assess the degrees of elemental homeostasis and imbalance to investigate whether sailfin catfishes could maintain elemental composition in face with variable dietary stoichiometry.

## 2 METHOD

### 2.1 Field study

**FIGURE 1:**
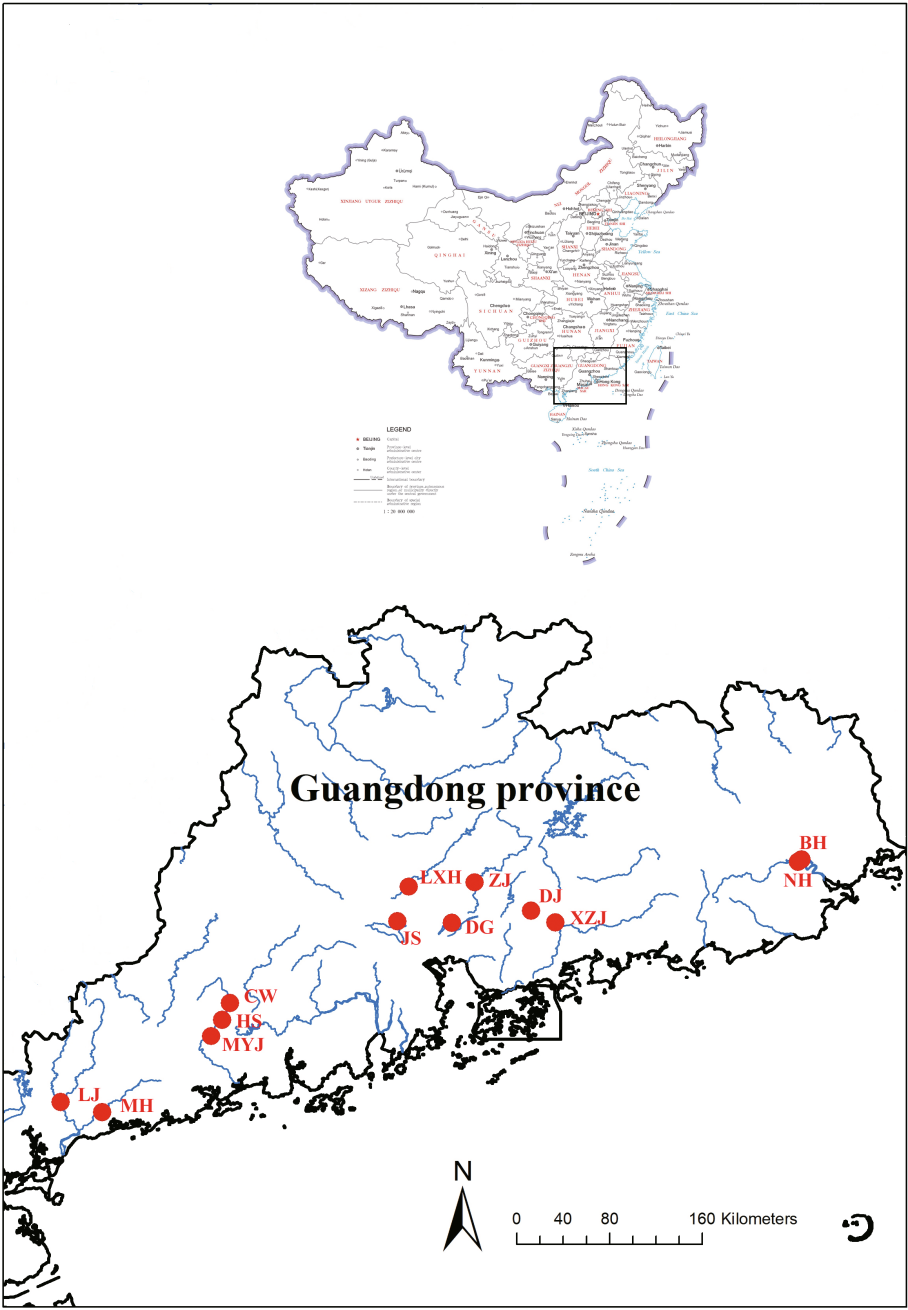
The map of sampling sites. The blue lines were rivers and the red symbols were sampling sites. The abbreviations of the sites indicated: BH, Beihe; CW, Chunwan; DG, Dongguan; DJ, Dongjiang; HS, Heshui; JS, Jiansha; LJ, Luojiang; LXH, Liuxihe; MH, Meihua; MYJ, Moyangjiang; NH, Nanhe; XZJ, Xizhijiang; ZJ, Zengjiang.

Specimens of sailfin catfishes were collected from 13 river sections across the main river basins of Guangdong province during late mid‐July and August in 2018, from East to West including Beihe (BH), Nanhe (NH), Xizhijiang (XZJ), Dongjiang (DJ), Zengjiang (ZJ), Dongguan (DG), Liuxihe (LXH), Jiansha (JS), Chunwan (CW), Heshui (HS), Moyangjiang (MYJ), Meihua (MH) and Luojiang (LJ) (Figure 1; Table S1). Guangdong province characterizes by a sub‐tropical monsoon climate with mean winter temperature of 13.3°C, mean summer temperature of 28.5°C, and mean annual rainfall of 1790 mm mainly between April and September (China Weather, 2018). These river sections were chosen to cover a range of environmental conditions, including differences in fish species richness, nutrient conditions, anthropic activity and climate (Li et al., 2013). The spatial heterogeneity can help to explore variation in stoichiometric traits and their relationship with environments.

Fish samples were collected by gill net and shrimp trap, then placed on ice immediately. A total of 70 specimens were collected to analyze the carbon (C), phosphorous (P) and calcium (Ca) contents of the whole individual from 13 river sections (See Table S1). Another 75 specimens were collected to investigate elemental allocations among bone, muscle and scute from 13 river sections (See Table S1). The standard length (nearest 0.01 mm) and total weight (nearest 0.01 g) of the specimens were measured to account for the covariation. All specimens were dissected to remove viscera. Sex was identified based on the morphology of the gonad. For the 75 specimens, all muscle was collected after removing the bone. Bones (i.e. vertebra and cranial bone) were scrubbed with a toothbrush and rinsed with deionized water to remove soft tissue. Bony scute (hereafter scute) was collected from the trunk and scrubbed to remove soft tissue.

To examine the relationship between environmental factors and stoichiometric traits of sailfin catfishes, data for biotic factors, climate, food quality and trophic status in the sampling sites were collected. The richness of fish species was estimated based on recent surveys in each river section (Wei et al., 2022). Climate data were sourced from the WorldClim database (records from 1970–2000) (Fick & Hijmans, 2017). Annual mean temperature (T), temperature seasonality (standard deviation ×100) (ΔT), annual precipitation (Rain) and precipitation seasonality (Coefficient of Variation) (ΔRain) were used in this study.

To assess the food quality in the habitats where sailfin catfishes were found, three in situ samples of detritus and periphyton were collected from each river section where the fishes were sampled. The aquatic sediment was collected from the river beds where sailfin catfishes were found by using a Peterson grab dredge (capacity = 5 L). The surface sediment (≈10 mm, mainly organic detritus and sand, hereafter detritus) was scraped by a plastic spoon. Periphyton (a mixture of detritus and algae) was gently scrubbed with a plastic brush from three to five stones and rinsed with deionized water into the acid‐washed plastic bottle. Food item in the foregut of three to six specimens in each site was squeezed into a 15 ml acid‐washed plastic tube (Hood et al., 2005).

To investigate the trophic status of the rivers, three in situ samples of seston and water samples were collected from each river section. Seston (mainly phytoplankton and detritus) was collected by a seston sampler (mesh size: 0.064 mm) and the slurry was transferred to an acid‐washed plastic bottle. Water samples were collected by a plastic water sampler (capacity = 5 L) to determine total nitrogen (TN) and phosphorous (TP), soluble reactive phosphorus (SRP) and ammonia nitrogen (NH_3_‐N) concentrations for each river section. The samples were placed in acid‐washed plastic bottles. Water samples for soluble reactive phosphorus and ammonia nitrogen analysis were filtered through glass‐fiber filters (Gelman A/E) to remove feces and other particles, then measured by the Multiparameter Meter (Lianhua Technology, Beijing, China) in the field. Water samples for total nitrogen and phosphorous analysis were acidified with 2 N H_2_SO_4_ (less than 2) and shipped to Pearl River Fisheries Research Institute, CAFS for analysis by the authors. The samples for TN analysis were digested with alkaline potassium persulfate under 122°C for 40 min and TP with potassium peroxodisulfate under 120°C for 30 min (State Environmental Protection Administration and Editorial Board of Water and Wastewater Monitoring and Analytical Methods, 2002). The solutions were measured by the Multiparameter Meter.

To measure C, P and Ca contents, all samples were dried at 60°C for about 48 h until a constant mass was achieved and maintained. Fish samples were homogenized and grounded to fine powder. The large particles of the detritus (>5 mm) were removed with forceps, then the detritus were homogenized and grounded into fine powder. The slurry samples of periphyton and seston were filtered onto pre‐ashed and pre‐weighed Whatman GF/F glass‐fiber filters and dried to determine C, P and Ca concentrations as per Datri et al. (2015). Carbon contents were analyzed by a TOC elemental analyzer (Vario TOC select, Elementar, Germany). Phosphorus and Ca were measured following the protocol of the Chinese national food safety standard for P and Ca measurement (GB 5009.87‐2016, 2016, GB 5009.92‐2016, 2016). Replicates of 0.2 ~ 3 g subsamples for P analysis were digested in mixed acid (i.e. H_2_SO_4_+ HNO_3_+ HClO_4_) at 120°C for 0.5 ~ 1 h, 180°C for 2 ~ 4 h and 200 ~ 220°C until the solution became transparent. The soluble P was measured based on the molybdenum blue method (State Environmental Protection Administration and Editorial Board of Water and Wastewater Monitoring and Analytical Methods, 2002). The subsamples (1 ~ 2 g) for Ca analysis were digested in mixed acid (i.e. HNO_3_+ HClO_4_) at 120 °C for 0.5 ~ 1 h, 180°C for 2 ~ 4 h and 200 ~ 220°C until the solution became transparent. The soluble Ca was measured using an EDTA titration method (Wuhan University, 2016). All element contents were expressed as % of dry weights and all elemental ratios as molar ratios.

### 2.2 Feeding experiment

A feeding experiment was conducted to investigate the impact of food quality on the stoichiometry of sailfin catfishes in a greenhouse at the Pearl River Fisheries Research Institute, CAFS, Guangzhou, China (23°04′ N, 113°13′ E) from 18 April to 29 May 2019. Twelve 100 L glass tanks were randomly assigned to the locations across the shelving units. Ten individuals of juvenile sailfin catfishes (between 75–90 days old) were kept in each tank in recirculation systems (temperature 25 ± 1°C). Aerated water, with oxygen, pH and hardness suitable for the fish, was provided from a central source tank. Treatment diets contained control, low, medium and high %P and %Ca. The basal diet was designed consulting the diet of the fish which is in the same trophic position as sailfin catfishes (Tables S2 and S3) (Jiang et al., 2014). Monocalcium phosphate [Ca(H_2_PO_4_)_2_] was added to manipulate P contents in the diet by consulting the threshold elemental ratio (TER_C:P_) of sailfin catfish, calculated as per (Frost et al., 2006) and the parameters of congeneric species. The ingredients were homogeneously mixed, air‐dried and compressed to pellets (size 1.5 mm) with a pelletizer. Dried food was kept in a freezer (−20°C) until used. Sailfin catfishes were fed to satiety twice daily for 42 days. Uneaten food was removed from each tank using a plastic pipette.

The total length (TL, nearest 0.01 mm) of each individual and total weight (TW, nearest 0.01 g) of ten individuals in each aquarium were measured at the beginning of the experiment. Total weight and liver weight (LW) of each individual were measured at the end of the experiment. The relative specific growth rate of total length (L‐SGR) was calculated using L‐SGR = (lnL_end_‐lnL_initial_)/t × L_initial_, with *L*_*initial*_ indicating the mean TL of the individuals in each aquarium at the beginning of the experiment, *L*_*end*_ indicated that at the end of the experiment and *t* indicated experimental period. The relative specific growth rate of total weight (W‐SGR) was calculated following *L‐SGR*. The relative growth of the liver, based on the hepatosomatic index was calculated as HSI = 100 × LW/TW at the end of the experiment. Three individuals in each tank were dried, grounded into powders and mixed as one sample to analyze C, P and Ca contents as per the *Field study*.

### 2.3 Statistical analysis

#### 2.3.1 Field study

##### The impacts of site identity and organism traits on body and organ stoichiometric traits of sailfin catfishes

The relationships of the standard length of sailfin catfishes to body and organ elemental contents and ratios were analyzed using Spearman correlation. Individual general linear models (GLM) were applied to assess the impact of site identity and sex on the variation of body and organ elemental contents and ratios of sailfin catfishes. The standard length was included in the model as a covariate. Data and residuals were tested for normality and data were transformed where needed. This statistical analysis was conducted under IBM SPSS statistics 19 platform.

##### The relationships among body and organ stoichiometric traits and environmental factors

Model selection based on general linear modeling combined with an information‐theoretic approach was conducted to investigate which environmental factors (i.e. biotic factor, climate, food quality, trophic status) could explain the variation in elemental contents and ratios in body, bone, muscle and scute of sailfin catfishes (Burnham & Anderson, 2002). The principal component of elemental contents and ratios in detritus, periphyton and seston were extracted using R package “FactoMineR”(Lê et al., 2008). The PCA parameter cos2, which indicated the contribution of a component the squared distance of the observation to the origin and the importance of a component for the observations in each site, was applied in further analysis. Before analysis, data were explored following the protocol of Zuur et al. (2010). Collinearity was checked using the variance inflation factor and a cut‐off value of 10 as a sign of collinearity as per Burnham and Anderson (2002). The initial screen also considered the biological meaning of environmental factors. For elemental content, climate factors (T and Rainfall), the first principle component axes for elemental contents in detritus, periphyton and seston (see Table S4), fish species richness and water chemistry (NH3‐N, TN and SRP) were selected. For elemental ratios, climate factors (T and ΔT), the first principle component axes for detritus, periphyton and seston (see Table S4), fish species richness and water chemistry (TN, TP and SRP) were selected. Spearman correlation analysis was also employed to exclude pairwise collinearity. There was no very strong relationship between the selected variables (*r* ≥ 0.7 or *r* ≥ −0.7), which indicated these environmental factors could be retained. All elemental data and environmental factors were Z‐score standardized.

Exhaustive screening of candidate sets of models was carried out with the R package “glmulti” (Calcagno & De Mazancourt, 2010), using all combinations of the main terms. Model selection was based on second‐order Akaike's information criterion (AICc) for the small sample size, with a minimum ΔAICc < 2 indicating relatively high‐level support of the selected models (Burnham & Anderson, 2002). The R package “MUMIN” was performed to average the top‐ranking models (i.e. ΔAICc < 2) (Bartoń, 2019).

##### Elemental imbalance between sailfin catfishes and diets in the field

Elemental imbalance was assessed as the arithmetic difference between diet and fish and calculated for body, bone, muscle and scute, respectively:

$${\text{Imbalance}}_{C:\text{Nutrient}}={\text{Diet}}_{C:\text{Nutrient}}–{\text{Catfish}}_{C:\text{Nutrient}}$$

Note that the arithmetic elemental imbalance is a coarse measurement and did not incorporate metabolism (i.e. loss of C in respiration). But it provided heuristic information to understand the trophic interaction between sailfin catfishes and resource quality in the field (Lauridsen et al., 2012). In this respect, positive imbalance values indicated the increased potential for nutrient (i.e. P and Ca) limitation, whereas negative values indicated the increased potential for C limitation.

##### The degree of stoichiometric homeostasis of sailfin catfishes in the feeding experiment

Individual GLMs were applied to examine the impact of food quality on elemental contents and ratios, W‐SGR, L‐SGR and HSI of sailfin catfishes. Body weight had no significant correlations with body elemental contents and ratios in the feeding experiment based on Spearman correlation (Table S5). Thus body weight was excluded from the model. All variables were the mean values for each aquarium in the analysis. Data were natural logarithm transformed to meet the assumption of normality where needed.

The degree of homeostasis was calculated by a linear equation with the variables logarithms transformed (Sterner & Elser, 2002):

$$\log\left(y\right)=\log\left(c\right)+\log\left(x\right)/H$$

c is a constant; 1/H is the slope of the regression between body (organs) and resources stoichiometry; y is the consumer stoichiometry and x is the diet stoichiometry. The degree of homeostasis was categorized as per Persson et al. (2010): (1) 1/H = 0 is “strictly homeostatic”, 0 < 1/H < 0.25 is “homeostatic”, 0.25 < 1/H < 0.5 is “weakly homeostatic”, 0.5 < 1/H < 0.75 is “weakly plastic” and 1/H > 0.75 is “plastic”.

## 3 RESULT

### 3.1 Field study

#### 3.1.1 General pattern

**FIGURE 2:**
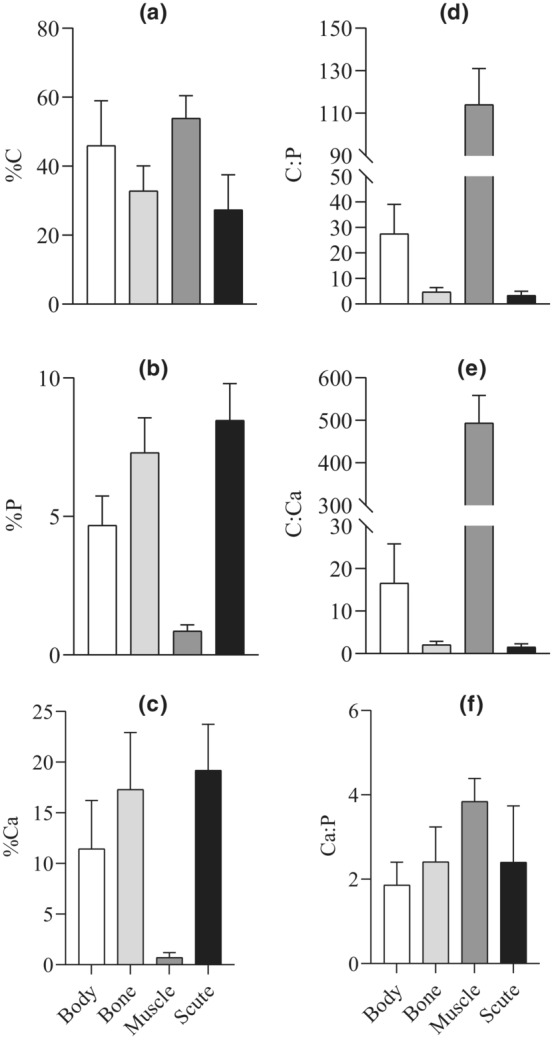
Body and organ elemental contents and ratios (mean ± SD) of sailfin catfishes *Pterygoplichthys* spp.

For the whole individual, %Ca was the most variable element, ranging from 5.05 to 22.49%, followed by %C (ranging from 27.18 to 72.46%) and then %P (ranging from 2.03 to 6.85%) across all sites (Figure 2a–c), leading to the variation of body C:P, C:Ca and Ca:P (Figure 2d–f). For organs, muscle %C was 1.64 fold of bone %C and 1.97 fold of scute %C across all sites (Figure 2a), resulting in relatively higher C:P and C:Ca in muscle (Figure 2d,e). Bone %C was 1.20 fold of scute %C (Figure 2a). Bone and scute %P were 8.46 fold and 9.81 fold of muscle, while bone and scute %Ca were 25.11 and 26.77 fold of scute muscle (Figure 2b,c), resulting in relatively lower C:P and C:Ca in bone and scute (Figure 2d,e). Scute %P and %Ca were 1.16 and 1.11 fold of bone (Figure 2b,c). Sailfin catfishes showed a wide range of variability in elemental contents and ratios in bone, muscle and scute (Figure 2). The variabilities of muscle %P and %Ca were greater relative to bone and scute (Figure 2b,c). The change of muscle %C was smaller relative to bone and scute (Figure 2a). Scute %P and %Ca had the least variability relative to other organs (Figure 2a–c).

#### 3.1.2 The impact of site identity and organism traits on stoichiometric traits

Site identity significantly affected body and organ elemental contents and ratios, except for scute %C, %Ca and C:Ca (Table 1). Body size had no significant effects on body and organ elemental contents and ratios (Table 1). Body and muscle elemental contents and ratios were not correlated with the standard length of sailfin catfishes (Table 2). Bone %P and %Ca and scute %P positively correlated with the standard length, while bone C:P and C:Ca and scute C:P negatively correlated with standard length (Table 2). Sex had a significant effect on body %C and C:Ca, while site*sex significantly affected bone C:P and C:Ca and muscle %P (Table 1). Males had more body %C and C:Ca than females (mean ± SD for %C: 50.54 ± 12.19 vs. 42.58 ± 12.61, and for C:Ca: 19.47 ± 9.84 vs. 14.37 ± 8.27).

**TABLE 1:** Results of individual general linear models (GLM) for the impacts of site identity (Site) and sex on the variation of body and organ elemental contents (carbon [%C], phosphorus [%P] and calcium [%Ca]) and ratios (C:P, C:Ca and Ca:P) in adult sailfin catfishes *Pterygoplichthys* spp. from field collections. The models included size as a covariate. The values in the table were *F* values.

Sample type	Variables	df	%C	%P	%Ca	ln(C:P)	ln(C:Ca)	ln(Ca:P)
Body	Size	1	0.03	0.03	0.11	0.034	0.163	0.24
	Site	12	3.22**	4.91***	12.31***	4.09***	5.94***	3.83***
	Sex	1	7.68**	0.01	1.707	3.345	5.42*	2.742
	Site*sex	10	0.53	0.47	0.5	0.637	0.705	0.903
Bone	Size	1	2.37	2.30	0.75	2.58	3.28	0.77
	Site	12	4.63***	6.67***	2.24*	6.47***	3.51*	4.24***
	Sex	1	0.04	0.04	0.08	0.00	0.06	0.14
	Site*sex	11	1.99	0.99	1.36	1.99*	2.17*	1.23
Muscle	Size	1	0.32	1.80	0.23	2.73	0.19	0.03
	Site	12	2.17*	7.32***	2.07*	5.63***	2.01*	2.79**
	Sex	1	0.03	0.03	0.01	0.00	0.03	0.05
	Site*sex	11	0.28	2.31*	0.92	1.76	0.92	0.99
Scute	Size	1	0.01	2.99	0.16	0.78	0.77	0.03
	Site	12	1.75	9.83***	0.67	3.63**	1.92	2.56*
	Sex	1	0.54	0.10	0.32	0.78	0.30	0.05
	Site*sex	11	0.89	1.16	1.27	0.63	1.00	0.92

*Note*: *p < .05, **p < .01, and ***p < .001.

**TABLE 2:** The Spearman correlation of body and organ elemental contents and ratios to the standard length of sailfin catfishes.

Variables	Standard length		
	*n*	*r*	*p*
Body			
%C	70	0.09	.47
%P	70	0.03	.79
%Ca	70	−0.07	.57
Ln(C:P)	70	0.03	.81
Ln(C:Ca)	70	0.09	.46
Ln(Ca:P)	70	−0.12	.30
Bone			
%C	75	−0.22	.06
%P	75	0.23	**.04**
%Ca	75	0.26	**.03**
Ln(C:P)	75	−0.24	**.04**
Ln(C:Ca)	75	−0.26	**.03**
Ln(Ca:P)	75	−0.05	.69
Muscle			
%C	75	0.00	.98
%P	75	−0.01	.96
%Ca	75	−0.17	.15
Ln(C:P)	75	0.02	.86
Ln(C:Ca)	75	0.19	.11
Ln(Ca:P)	75	−0.19	.11
Scute			
%C	75	−0.16	.17
%P	75	0.35	**.00**
%Ca	75	0.10	.42
Ln(C:P)	75	−0.31	**.01**
Ln(C:Ca)	75	−0.15	.21
Ln(Ca:P)	75	−0.15	.20

*Note*: The bold text indicated a significant relationship.

#### 3.1.3 The relationships between body and organ stoichiometric traits and environmental factors

**FIGURE 3:**
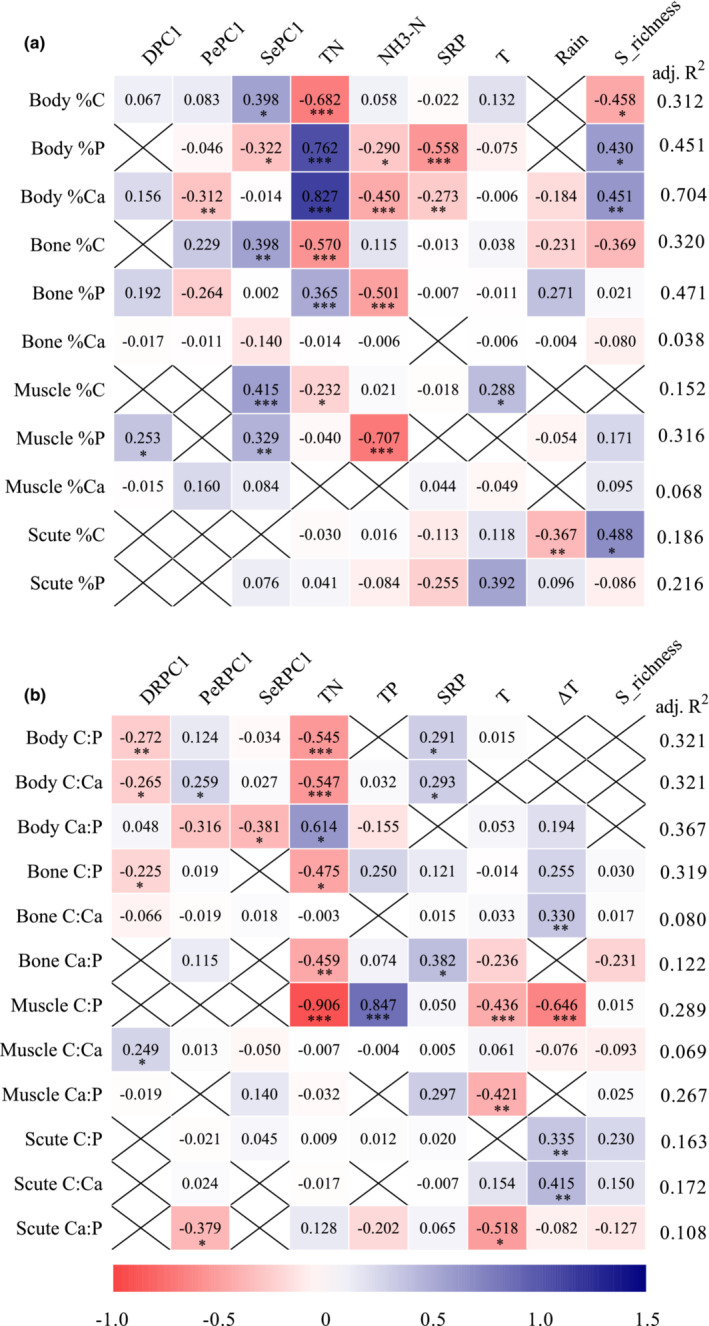
Model selection results for the relationship of environmental factors to elemental contents (a) (i.e. carbon [%C], phosphorus [%P] and calcium [%Ca]) and ratios (b) (i.e. C:P, C:Ca and Ca:P) in body, bone, muscle and scute of sailfin catfishes *Pterygoplichthys* spp. The cross indicated the factor excluded from the best model (ΔAICc<2). The values in the squares indicated average parameter estimates (standardized regression coefficients) of the model predictors. The adjusted (adj.) R^2^ of the best model was given on the right and the significance of each predictor were given as: *p < .05; **p < .01; ***p < .001. The abbreviations of environmental factors indicated: DPC1, PC1 for detritus elemental contents; DRPC1, PC1 for detritus elemental ratios; NH3‐N, ammonia‐nitrogen; PePC1, PC1 for periphyton elemental contents; PeRPC1, PC1 for periphyton elemental ratios; Rain, annual precipitation and S_richness, fish species richness; SePC1, PC1 for seston elemental contents; SeRPC1, PC1 for seston elemental ratios, TN, total nitrogen concentration in the rivers; SRP, soluble reactive phosphorus; T, Annual mean temperature; TP, total phosphorus concentration in the rivers; ΔT, temperature seasonality (standard deviation ×100).

The variation between elemental contents and ratios of sailfin catfishes could be explained by a combination of climate, diet quality, fish species richness, and trophic status of the rivers (Figure 3a,b). Total nitrogen could explain more than 50% of the variation in body elemental contents and ratios, bone %C and muscle C:P (Figure 3a,b). In this respect, TN negatively related with body %C, C:P and C:Ca and muscle C:P, while had a positive relationship with body %P, %Ca and Ca:P (Figure 3a,b). NH3‐N could explain more than 50% of the variation of bone %P and muscle %P, and the regression was negative (Figure 3a). TP could explain 84.7% of the variation of muscle C:P and the regression was positive (Figure 3b). SRP could explain 55.8% of the variation of body %P and the regression was negative (Figure 3a). Annual mean temperature could explain 51.8% of the variation in scute Ca:P and the regression was negative (Figure 3b). Temperature seasonality could explain 64.6% of the variation in muscle C:P and the regression was negative (Figure 3b). Although there were significant relationships among several environmental factors (e.g. Rain, diet quality, seston quality, fish species richness, etc.) and stoichiometric traits of body and organs, these environmental factors only could explain lower than 50% of the variation (Figure 3a,b), which indicated relatively weak relationships between body and organ stoichiometric traits and these environmental factors.

#### 3.1.4 Elemental imbalance between sailfin catfishes and diets in the field

**FIGURE 4:**
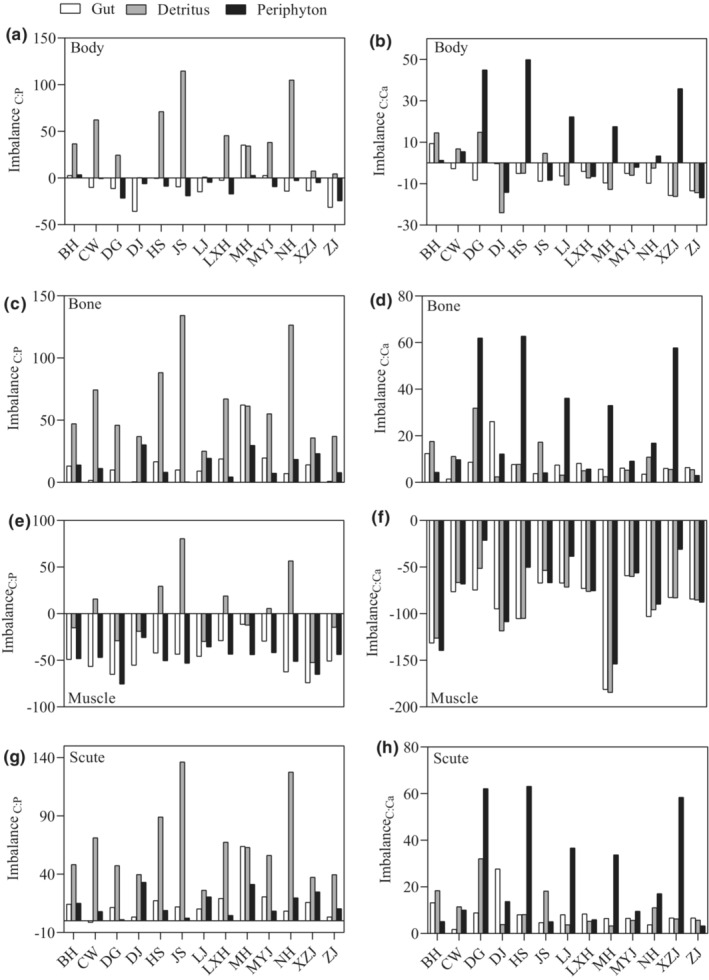
Arithmetic elemental imbalances between body (organs) of sailfin catfishes *Pterygoplichthys* spp. and diets (diets in the foregut, entire detritus and entire periphyton) in different sites. See Figure 1 for the abbreviation information of sampling sites.

Elemental contents and ratios of detritus varied significantly among sites, while only %P, C:P and C:Ca of periphyton showed significantly spatially differences (Table S6). Also, elemental imbalances varied among sites and diet categories (Figure 4). Arithmetic imbalance value between diet and body C:P was generally negative based on periphyton and diet in the foregut, while positive based on detritus (Figure 4a). In this respect, sailfin catfishes were likely to undergo C limitation when assumed based on periphyton and diet in the foregut and might experience P limitation when assumed based on detritus. The arithmetic imbalance between diet and body C:Ca was generally negative based on the diet in foregut and detritus, and generally positive based on periphyton (Figure 4b). In this respect, sailfin catfishes were likely to undergo C limitation based on detritus and diet in the foregut, while they might experience Ca limitation based on periphyton in some sites. Elemental imbalances also varied among organs. Arithmetic imbalances between all diet categories and bone and scute C:P and C:Ca were positive (Figure 4c,d,g,h), which indicated P and Ca limitation, whereas imbalance values between all diet categories and muscle were negative, indicating C limitation (Figure 4e,f). Muscle C:P and C:Ca were generally more imbalanced relative to the body, bone and scute (Figure 4).

#### 3.1.5 The degree of stoichiometric homeostasis of sailfin catfishes in the feeding experiment

**FIGURE 5:**
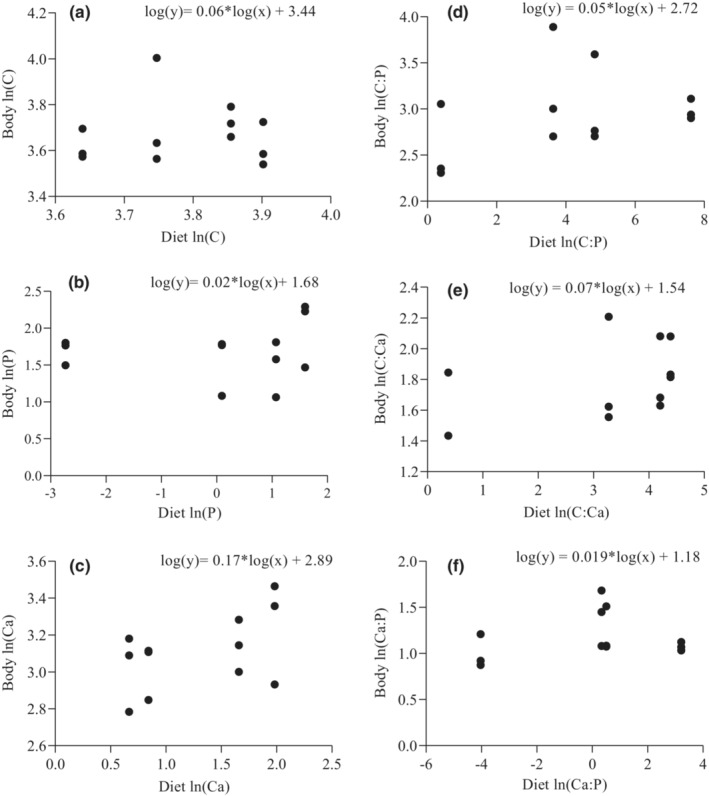
The degree of elemental homeostasis of sailfin catfishes *Pterygoplichthys* spp. The slopes of regression between body and diet stoichiometry in the feeding experiment were used to assess the degree of elemental homeostasis by the following: (1) 1/H = 0 is “strictly homeostatic”, 0 < 1/H < 0.25 is “homeostatic”, 0.25 < 1/H < 0.5 is “weakly homeostatic”, 0.5 < 1/H < 0.75 is “weakly plastic” and 1/H > 0.75 is “plastic”.

Sailfin catfishes exhibited strict homeostasis in the experiment. In this respect, food quality had no significant effects on body elemental contents and ratios, as well as the growth of sailfin catfishes (Table 3). Also, there was no significant relationship in elemental contents and ratios between sailfin catfishes and their diets (Figure 5). Sailfin catfishes exhibited stoichiometric homeostasis (0 < 1/H < 0.25) for all elemental contents and ratios (Figure 5a–f) and the highest 1/H was found in the relationship between body and diet %Ca (Figure 5c).

**TABLE 3:** General linear model results for the impacts of Monocalcium phosphate [Ca(H_2_PO_4_)_2_] addition in the diet (Diet) on elemental content (i.e. %C, %P and %Ca), and ratios (i.e. C:P, C:Ca and Ca:P), the relative specific growth rate of body weight (W‐SGR) and total length (L‐SGR) and hepatosomatic index (HSI) of sailfin catfishes *Pterygoplichthys* spp.

Response variables	Effect	df	*F*	*p*
%C	Diet	3,12	0.66	.60
%P	Diet	3,12	1.64	.26
%Ca	Diet	3,12	1.07	.41
Ln(C:P)	Diet	3,12	1.04	.43
Ln(C:Ca)	Diet	3,12	0.90	.48
Ln(Ca:P)	Diet	3,12	2.01	.19
L‐SGR	Diet	3,12	0.58	.65
W‐SGR	Diet	3,12	0.31	.82
HSI	Diet	3,12	2.08	.18

## 4 DISCUSSION

### 4.1 General pattern

Although classical stoichiometric theory proposed that animals could maintain fixed elemental contents and ratios, recent studies demonstrated intraspecific variation in stoichiometric traits of animals due to their adaptive response to context‐specific environments in the habitats (Snell‐Rood et al., 2015; Sterner & Elser, 2002). The results of this study could support the later perspective that sailfin catfishes exhibited considerable variability in elemental contents and ratios in the body and organs across the populations. Site identity, which can represent the complexity of environments in the habitats, was the main factor contributing to stoichiometric trait variation. This effect could be explained by the significant relationships between stoichiometric traits and a combination of environmental factors (i.e. trophic status, diet quality, climate and richness of fish species), suggesting these environmental factors were the main driver of the diversity of stoichiometric traits across different geographical regions.

### 4.2 The impacts of organism traits on the variation of stoichiometric traits

On the other hand, body size and sex had no significant impacts on body elemental contents, except for body %C. In this regard, male body %C was higher than female, which was not consistent with the previous study for *Lepomis gibbosus* and *Perccottus glenii* (Mozsar et al., 2019). Males of both species exhibited nest‐guarding activities, resulting in a decline in %C due to food deprivation and energy reserves decrease (Pizzolon et al., 2012). For sailfin catfishes, males have tremendous investments in burrowing nests before females spawn and their body condition could recover after females spawn (Gibbs et al., 2017; Lienart et al., 2013). These differences might explain the lower %C in females relative to males in this study which were collected during or after female spawning in August (Gibbs et al., 2017). It is also possible that females allocate more %C to nutrient‐rich reproductive tissues during spawning. Positive correlation between body size and %P and %Ca in bone and scute suggested allometry of %P and %Ca investment in bone and scute. It was consistent with stoichiometric theory predictions that %P increased with body size due to a greater allocation in P‐rich bony tissues for vertebrates (Sterner & Elser, 2002), whereas body elemental contents remained constant with the variation of body sizes, which suggested changes in elemental allocation among organs had little impact on body stoichiometric traits (Jeyasingh et al., 2014). Besides, allometric relationships of %P and %Ca between organs of sailfin catfishes (see Table S7) manifested elemental allocation among organs at different rates (Hendrixson et al., 2007). This pattern was likely to lead to more allocation of %P and %Ca in the scute relative to the bone, owing to differences in anatomical structures between bone and scute (Soliman et al., 2020). These results promoted the understanding that organismal traits could affect the variation of intraspecific stoichiometric traits owing to divergent nutrient demands of fish in different body sizes, sexes and morphologies (Burress et al., 2013; Durston & El‐Sabaawi, 2017; Pilati & Vanni, 2007).

### 4.3 The relationships among body and organ stoichiometric traits and environmental factors

Stoichiometric traits were more sensitive to the water chemistry of the rivers (i.e. TN, TP, NH3‐N and SRP) relative to diet quality, climate and biotic factors. Water chemistry had direct and indirect impacts on aquatic species by providing nutrients (Allgeier et al., 2018). In this study, TN had negative relationships with %C and C:nutrient in the body and organs of sailfin catfishes, which might attribute to nutrient imbalance (e.g. C limitation) between fish and basal resources under high N habitats (Penuelas et al., 2013). The increase of body and organ %P and %Ca with the increase of TN could be explained by the decrease of %C in high TN conditions. In this regard, body and organ %C were negatively correlated with %P and %Ca, as the increase of %C in tissues could “dilute” the %P and %Ca of fish body and tissues (Boros et al., 2015; Durston & El‐Sabaawi, 2017; Pilati & Vanni, 2007). The negative relationship between SRP and body %P and %Ca, as well as the positive relationship between SRP and body C:P and C:Ca might be related to low P and Ca excretion rate and high P acquisition due to high P and Ca demand of sailfin catfishes for growth and skeletogenesis (Hood et al., 2005; Moody et al., 2019; Zandonà et al., 2020). Besides, water chemistry could affect aquatic species due to biological toxicity (Randall & Tsui, 2002). Increasing ammonium and nitrate in the waters could lead to adverse physiological and histopathological reactions in fish (Brinkman et al., 2009), in turn, resulting in a negative effect on the growth performance and maturity of the fishes (Wei et al., 2022). Elevated NH3‐N could cause excessive activation of NMDA receptor, resulting in an influx of surplus intracellular Ca^2+^ from Ca‐rich cells and cell death in the central nervous system (Randall & Tsui, 2002). In this regard, the negative relationship between NH3‐N and organ %P and %Ca might be related to NH3‐N induced biochemical reactions in the cells of different tissues (e.g. Randall & Tsui, 2002). These results suggested inorganic nutrient mediated direct and indirect reactions of fishes to nutrient imbalance and ammonia toxicity might play an important role in regulating stoichiometric variation of non‐native fishes across different rivers.

Climate is one of the critical sources of intraspecific variation of stoichiometric traits, which might be driven by climate‐related changes in water pH and nutrient availability (Hasler et al., 2018; Junker & Cross, 2014). In this respect, warm temperature might decrease calcium carbonate minerals in the biological structures of organisms due to acidification as a result of increases in carbon dioxide dissolution (Hasler et al., 2018; Leung et al., 2022). These findings might explain the decrease of scute Ca:P with the annual mean temperature increase. Besides, variation in stream temperature could affect the quantity and quality of food resources due to the input of terrestrially derived nutrients (e.g. leaf litter C) (Junker & Cross, 2014). In this respect, the changes in diet quantity and quality might lead to the variation in nutrient imbalance (i.e. P and Ca limitation) between consumer and resource (Halvorson et al., 2017). These changes might be related to the increase of C:P and C:Ca in bone and scute. Besides, temperature variation could also change the feeding behavior of consumers to search for suitable resources (Guzzo et al., 2017). These changes might lead to high P absorption to support ATP (i.e. P‐rich biomolecule) requirement in locomotive organs (e.g. muscle) (Allen & Trajanovska, 2012), resulting in lower muscle C:P. Further studies were needed to investigate the mechanism underpinned the impact of climate‐related changes driven by nutrient imbalance on animals' performance, which could provide new insight into the understanding of the adaption of stoichiometric traits under climate change conditions.

### 4.4 How did sailfin catfishes handle elemental imbalance in the invaded rivers?

Animals can maintain elemental composition constantly through a series of physiological processes in face with dynamic food quality (Sterner & Elser, 2002). However, studies suggested that animals may be less homeostatic in their stoichiometry than previously thought. For example, recent studies demonstrated that stream insect consumers and a planktonic rotifer could not maintain strict homeostasis at high or low resource P conditions (Small & Pringle, 2010). Similarly, the degree of P homeostasis of fishes varied from species to species (i.e. strict vs weak homeostasis) (Persson et al., 2010). The elemental content of fishes could be affected by ontogenetic‐related diet shift and diet selection in different habitats (Pilati & Vanni, 2007; Vrede et al., 2011). Besides, diet quality could also explain the variation of fish stoichiometric traits, especially for P (Dalton et al., 2017; El‐Sabaawi, Kohler, et al., 2012; El‐Sabaawi, Zandona, et al., 2012). In this study, juvenile sailfin catfishes can maintain stoichiometric homeostasis, since elemental contents and ratios of sailfin catfishes from the feeding experiment were not changed as diet stoichiometry. Albeit weak, in the field, the changes in elemental contents and ratios of sailfin catfishes were related to diet quality. These inconsistent findings could be explained by Halvorson and Small (2016) that field observations were not applicable to assess consumer stoichiometric homeostasis owing to the covariation of confounding factors. Fishes from different rivers might experience different evolution histories as a result of adapting to local environmental conditions (e.g. resource availability, predation regime, etc.), which might lead to different stoichiometric related traits (e.g. body size, metabolic rate, etc.) (El‐Sabaawi et al., 2014). For example, the diet compositions of sailfin catfishes varied in different habitats according to the availability of the food supply in the habitats (Lujan et al., 2012, H, Wei personal observation). In this study, sailfin catfishes experienced different degrees of P and Ca limitation when feeding on different diets across different sites. For this reason, stoichiometric traits of fishes from different rivers might be more variable than that of fishes in the manipulation experiment (i.e. manipulating the same environmental condition and fish body size) in response to the changes in diet quality. In this study, the imbalances between sailfin catfishes and diets differed among sampling sites and diet categories, which could result in the spatial variation of stoichiometric traits of sailfin catfishes. Those results, jointly indicated that non‐native fishes could change the stoichiometric traits to handle dynamic diet quality in the invaded rivers.

## 5 CONCLUSION

In this study, sailfin catfishes can exhibit considerable variation in stoichiometric traits in response to the environmental dynamics of the invaded rivers. Site identity was the main factor contributing to the variation, which could be explained by a combination of environmental factors including climate, diet quality, fish species richness and trophic status in the invaded rivers. Water chemistry contributed to the most variation of stoichiometric traits of sailfin catfishes, which might be mediated by the direct and indirect response of the fishes to nutrient imbalance and ammonia toxicity in the habitats. Imbalances of P and Ca between sailfin catfishes and food resources varied among sampling sites, reflecting the spatial heterogeneity of nutrient limitation. These findings suggested variation in stoichiometric traits might be attributed to the changes in elemental metabolism to cope with context‐specific environments which directly or indirectly affected consumer performance, food resource quality and elemental imbalance (Leal et al., 2017b). Those changes could promote non‐native fishes to acclimatize to context‐specific environments in invaded rivers (Zandonà et al., 2020). This study provided heuristic knowledge about environmental‐related variation in stoichiometric traits, which could enhance the understanding of the non‐native species' adaptation to resource fluctuation in the invaded ecosystems.

## AUTHOR CONTRIBUTIONS

**Hui Wei:** Conceptualization (lead); data curation (equal); formal analysis (equal); funding acquisition (equal); investigation (equal); methodology (equal); validation (equal); writing – original draft (lead). **Yanting Liang:** Data curation (equal); investigation (equal); writing – review and editing (equal). **Qiang Luo:** Data curation (equal); investigation (equal); writing – review and editing (equal). **Dangen Gu:** Formal analysis (equal); funding acquisition (equal); investigation (equal); writing – review and editing (equal). **Xidong Mu:** Data curation (equal); investigation (equal); methodology (equal); writing – review and editing (equal). **Yinchang Hu:** Conceptualization (equal); formal analysis (equal); project administration (equal); supervision (equal); validation (equal); visualization (equal); writing – review and editing (equal).

## CONFLICT OF INTEREST

The authors have no conflict of interest to declare.

## Supporting information


**Appendix S1** Supporting Information

## Data Availability

The data are available at DOI https://doi.org/10.5061/dryad.8pk0p2npk.
