# Supplementary material for: Environmental‐related variation of stoichiometric traits in body and organs of non‐native sailfin catfishes Pterygoplichthys spp
Source: Ecol Evol. 2022 Nov 5;12(11):e9483. doi: 10.1002/ece3.9483 (PMC9636514; doi:10.1002/ece3.9483)
Supplement: Supplementary file 1 — Appendix S1 Supporting Information [file ECE3-12-e9483-s001.docx]

Table S 1 The coordinates, number of specimens for body (n1) and organ (n2) stoichiometric analysis, the body weight range of the specimens.

| River | Abbreviation | Longtitude | Latitude | n1 | Size range (g) | n2 | Size range (g) |
| --- | --- | --- | --- | --- | --- | --- | --- |
| Beihe River | BH | 116.4 | 23.5 | 4 | 234.47-329.42 | 5 | 249.33-465.9 |
| Chunwan River | CW | 111.9 | 22.4 | 6 | 88.58-228.23 | 2 | 166.57-206.02 |
| Dongguan Rive | DG | 113.7 | 23.1 | 6 | 240.73-363.01 | 6 | 236.93-381.19 |
| Dongjiang River | DJ | 114.3 | 23.2 | 5 | 184.1-369.17 | 6 | 179.68-366.6 |
| Heshui River | HS | 111.9 | 22.3 | 3 | 214.77-387.49 | 8 | 214.77-497.54 |
| Jiansha River | JS | 113.2 | 23.1 | 6 | 305.81-549.75 | 6 | 146.67-398.63 |
| Luojiang River | LJ | 110.6 | 21.7 | 6 | 201.02-356 | 7 | 216.6-352.35 |
| Liuxihe River | LXH | 113.3 | 23.3 | 6 | 123.56-243.78 | 6 | 176.79-280.99 |
| Meihuajiang River | MH | 110.9 | 21.6 | 6 | 120.26-226.43 | 5 | 162.26-234.96 |
| Moyangjaing River | MYJ | 111.8 | 22.2 | 4 | 164.2-269.91 | 6 | 187.97-236.85 |
| Nanhe River | NH | 116.3 | 23.5 | 6 | 130.9-488.61 | 6 | 177.41-339.9 |
| Xizhijiang River | XZJ | 114.5 | 23.1 | 6 | 149.51-302.05 | 6 | 121.96-263.91 |
| Zengjiang River | ZJ | 113.8 | 23.4 | 6 | 160.02-323.31 | 6 | 162.42-290.31 |

Table S2 Formulation and proximate composition (% air-dry basis) of the basal diet.

| Ingredients | % |
| --- | --- |
| Casein | 30 |
| Gelatin | 7.5 |
| Corn starch | 38 |
| Corn oil | 4 |
| Soybean oil | 4 |
| Choline chloride | 0.25 |
| Cellulose | 11.25 |
| Premix | 1 |

Note: Premix provided the following minerals: Ca( CH_3_CHOHCOO)_2_·5H_2_O 327 g，FeSO_4_·6H_2_O 2.125 g, MgSO_4_ 7H_2_O 137 g, NaCl 43.5 g, AlCl_3_·6H_2_O 0.15 g, KIO3 0.125 g, KCl 75 g, CuCl_2_·2H_2_O 0.1 g, MnSO_4_·H_2_O 0.80 g, CoCl_2_·6H_2_O 1 g and ZnSO_4_·7H_2_O 3 g.

Table S3 The amount of monocalcium phosphate [Ca(H_2_PO_4_)_2_] and the corresponding total phosphorus and calcium in the artificial feeds with four gradients of Ca(H_2_PO_4_)_2_ additions.

| Treatment | Amounts of Ca(H_2_PO_4_)_2_（%） | %C | SD | %P | SD | %Ca | SD |
| --- | --- | --- | --- | --- | --- | --- | --- |
| Control | 0 | 47.23 | 5.16 | 0.07 | 0.02 | 1.95 | 0.08 |
| Low | 1.7 | 49.49 | 3.54 | 1.10 | 0.19 | 2.32 | 0.03 |
| Medium | 13.6 | 42.41 | 3.34 | 2.91 | 0.04 | 5.25 | 0.49 |
| High | 27.2 | 38.07 | 3.08 | 4.93 | 0.05 | 7.26 | 0.04 |

**Instruction for Table S4:**

**Principle component analysis for elemental contents and ratios in detritus, periphyton and seston**

*Statistic analysis*

A principle component analysis (PCA) was conducted to reduce the dimension of elemental contents (i.e. %C, %P and %Ca) and ratios (i.e. C:P, C:Ca and Ca:P) in detritus, periphyton and seston respectively by using R package “FactoMineR”(Lê et al., 2008). The first two principle component axes were used to investigate the relationships between elemental contents and ratios of detritus, periphyton and seston and stoichiometric traits of sailfin catfishes. The PCA parameter cos2, which indicated the contribution of a component the squared distance of the observation to the origin and the the importance of a component for the observations in each site, was applied in further analysis.

*Results*

Principle component analysis indicated that DPC1 accounted for 83% of the variation of elemental content, reflected positive relationship between PC1 and elemental contents (i.e. %C, %P and %Ca) (Table 3). PePC1 contributed to 51.3% of the variation, indicated PC1 had a positive relationship with %P and %Ca content and negative relationship with %C. SePC1 contributed to 46.6% of the variation, reflected PC1 had a negative relationship with %C and positive relationship with %Ca. DRPC1 contributed to 54.3% of the variation of elemental ratios (Table 3), reflected PC1 had a negative relationship with C:Ca and positive with Ca:P. PeRPC1 of elemental ratios contributed 57.2% of the variation, indicated PC1 had a negative relationship with C:Ca and positive relationship with Ca:P. SeRPC1 contributed 61.7% of the variation, indicated PC1 had a positive relationship with C:Ca and Ca:P. Elemental contents and ratios of detritus varied significantly amongst sites, while only P%, C:P and C:Ca of periphyton significantly differed amongst sites (Table S6).

Table S4 Factor loadings of principle component analysis for elemental contents (carbon [%C], phosphorus [%P] and calcium [%Ca]) and ratios (C:P, C:Ca and Ca:P) in detritus, periphyton and seston. Significant correlation (value ≥ 0.5) between variable and principle component axis was highlighted in bold.

| Factor | PC1 | PC2 | PC3 |
| --- | --- | --- | --- |
| Detritus C | **0.98** | -0.03 | -0.19 |
| Detritus P | **0.88** | -0.45 | 0.12 |
| Detritus Ca | **0.86** | 0.49 | 0.10 |
| Cumulative variance | 83% | 98% | 100% |
| Periphyton C | **-0.63** | **0.69** | 0.35 |
| Periphyton P | **0.88** | 0.00 | 0.47 |
| Periphyton Ca | **0.60** | **0.73** | -0.33 |
| Cumulative variance | 51% | 85% | 100% |
| Seston C | **-0.79** | 0.16 | **0.59** |
| Seston P | 0.45 | **0.89** | 0.12 |
| Seston Ca | **0.75** | -0.36 | 0.55 |
| Cumulative variance | 47% | 78% | 100% |
| Detritus C:P | 0.38 | **0.90** | -0.20 |
| Detritus C:Ca | **-0.75** | **0.61** | 0.24 |
| Detritus Ca:P | **0.96** | 0.12 | 0.27 |
| Cumulative variance | 54% | 94% | 100% |
| Periphyton C:P | 0.28 | **0.95** | -0.16 |
| Periphyton C:Ca | **-0.86** | 0.42 | 0.29 |
| Periphyton Ca:P | **0.95** | 0.10 | 0.31 |
| Cumulative variance | 57% | 93% | 100% |
| Seston C:P | **0.60** | **0.78** | -0.17 |
| Seston C:Ca | **0.96** | 0.00 | 0.27 |
| Seston Ca:P | **-0.75** | **0.63** | 0.21 |
| Cumulative variance | 62% | 95% | 100% |

Table S5 Spearman correlation between total length and body elemental contents and ratios of sailfin catfishes in feeding experiment

| Variables |  | Standard Length | |
| --- | --- | --- | --- |
|  | n | r | p |
| %C | 12 | 0.13 | 0.69 |
| %P | 12 | -0.14 | 0.66 |
| %Ca | 12 | -0.31 | 0.32 |
| Ln(C:P) | 12 | 0.22 | 0.50 |
| Ln(C:Ca) | 12 | 0.34 | 0.29 |
| Ln(Ca:P) | 12 | 0.14 | 0.66 |

Table S6 Elemental contents and ratios of basal resources variation in different sites

| Elemental composition | df | Detritus | | Seston | | Periphyton | |
| --- | --- | --- | --- | --- | --- | --- | --- |
|  |  | F | P | F | P | F | P |
| %C | 12 | 14.87 | **<0.001** | 5.46 | **<0.001** | 1.24 | 0.31 |
| %P | 12 | 7.40 | **<0.001** | 2.93 | **0.01** | 2.75 | **0.02** |
| %Ca | 12 | 4.89 | **<0.001** | 4.32 | **0.00** | 1.69 | 0.13 |
| ln(C:P) | 12 | 5.81 | **<0.001** | 5.38 | **<0.001** | 2.69 | **0.02** |
| ln(C:Ca) | 12 | 3.13 | **0.01** | 6.26 | **<0.001** | 2.15 | **0.05** |
| ln(Ca:P) | 12 | 3.10 | **0.01** | 4.75 | **<0.001** | 1.79 | 0.10 |

**Instruction for Table S7:**

**Elemental trade-off between organs**

*Statistic analysis*

Standardized major axis (SMA) analyses were applied to test the allometric scaling relationships of %P and %Ca in different organs using R package “smatr” . The regression curve was expressed by the equation: log Y = a*log X + b, where X and Y represented the %P or %Ca in different organs, a is the slope (i.e. scaling exponent) and b is the intercept of the regression.

*Results*

Standardized major axis analyses revealed that the scaling slopes of the relationships of %P and %Ca between muscle and bone, between scale and bone, as well as between muscle and scale were different from 1 with 95% confidence intervals, which indicated allometric relationships (P<0.001) ( Table S 7).

Table S7 The parameters of scaling relationship of %P/%Ca in different organs. The differences between scaling slope and 1 were estimated by coverage of the 95% confidence interval.

| Organ elemental content | Scaling slope (a) | 95% CI | R^2^ | *P* | Test for allometry *P* |
| --- | --- | --- | --- | --- | --- |
| Bone vs Muscle |  |  |  |  |  |
| P | 1.54 | 1.54- 1.91 | 0.17 | <0.001 | <0.001 |
| Ca | 2.75 | 2.18 - 3.48 | 0.00 | 0.78 | <0.001 |
| Bone VS Scute |  |  |  |  |  |
| P | 0.77 | 0.68-0.87 | 0.71 | <0.001 | <0.001 |
| Ca | 0.69 | 0.55 - 0.85 | 0.18 | <0.001 | <0.001 |
| Muscle vs Scute |  |  |  |  |  |
| P | 2.00 | 1.64 - 2.45 | 0.26 | <0.001 | <0.001 |
| Ca | -4.01 | -5.05--3.18 | 0.03 | 0.13 | <0.001 |
